# Linking stroke mortality with air pollution, income, and greenness in northwest Florida: an ecological geographical study

**DOI:** 10.1186/1476-072X-7-20

**Published:** 2008-05-01

**Authors:** Zhiyong Hu, Johan Liebens, K Ranga Rao

**Affiliations:** 1 Department of Environmental Studies, University of West Florida, Pensacola, FL, USA; 2 Center for Environmental Diagnostics and Bioremediation, University of West Florida, Pensacola, FL, USA

## Abstract

**Background:**

Relatively few studies have examined the association between air pollution and stroke mortality. Inconsistent and inclusive results from existing studies on air pollution and stroke justify the need to continue to investigate the linkage between stroke and air pollution. No studies have been done to investigate the association between stroke and greenness. The objective of this study was to examine if there is association of stroke with air pollution, income and greenness in northwest Florida.

**Results:**

Our study used an ecological geographical approach and dasymetric mapping technique. We adopted a Bayesian hierarchical model with a convolution prior considering five census tract specific covariates. A 95% credible set which defines an interval having a 0.95 posterior probability of containing the parameter for each covariate was calculated from Markov Chain Monte Carlo simulations. The 95% credible sets are (-0.286, -0.097) for household income, (0.034, 0.144) for traffic air pollution effect, (0.419, 1.495) for emission density of monitored point source polluters, (0.413, 1.522) for simple point density of point source polluters without emission data, and (-0.289,-0.031) for greenness. Household income and greenness show negative effects (the posterior densities primarily cover negative values). Air pollution covariates have positive effects (the 95% credible sets cover positive values).

**Conclusion:**

High risk of stroke mortality was found in areas with low income level, high air pollution level, and low level of exposure to green space.

## Background

Stroke is a type of cardiovascular disease that affects the arteries leading to and within the brain. A stroke occurs when a blood vessel that carries oxygen and nutrients to the brain is either blocked by a clot (ischemic stroke) or bursts (hemorrhagic stroke) [1]. When that happens, part of the brain cannot get the blood (and oxygen) it needs, so it starts to die. It is the third leading cause of death in the Western world, after heart disease and cancer [2]. Stroke could soon be the most common cause of death worldwide [3]. The chances of having a stroke more than double for each decade of life after age 55 [4,5]. Stroke is more common in men than in women [5]. According to the American Heart Association, African Americans have a higher risk of stroke than Caucasians do [6]. While a number of risk factors for stroke, e.g., age, gender, and ethnicity, are well known, the potential importance of outdoor air pollution is much less recognized. Other less well documented risk factors include socioeconomic factors and physical inactivity.

Air pollution is known to be associated with cardiovascular disease, but relatively few studies have examined the association between air pollution and stroke mortality [7]. There is increasing evidence linking outdoor air pollution and stroke [7-11] using either ambient air pollutant concentration data or proximity to polluters as a surrogate exposure. A study conducted in Seoul, Korea, has shown that commonly measured pollutants (O_3_, SO_2_, NO_2_, CO, PM_10_) are all significantly associated with stroke mortality [7]. In a study conducted in the Netherlands, gaseous air pollutants (O_3_, CO, SO_2_) were found to be significantly associated with stroke mortality [12]. In Hong Kong, however, none of 4 pollutants (SO_2_, NO_2_, O_3_, PM_10_) studied were found to be significantly associated with stroke mortality [13]. Using hospital admission records from Taiwan, Tsai [9] found a statistically positive association between PM_10 _and both ischemic and hemorrhagic strokes. In a small-area level ecological study, Maheswaran [11] found that stroke incidence rates are higher in more polluted areas due to the combined effects of acute and chronic exposure. Maheswaren and Elliott [14] examined the association between stroke mortality and air pollution at the small-area level using proximity to main roads as a proxy for exposure to road traffic pollution, and a 5% increase in stroke mortality was observed in areas within 200 m of a main road [14]. Inconsistent and inclusive results from existing studies justify the need to continue to investigate the association between stroke and air pollution.

There is considerable evidence of income inequality affecting health [15,16]. More and more studies have examined how income inequality affects health. Marmot [17] discussed two ways in which income could be causally related to health: through a direct effect on the material conditions necessary for biological survival, and through an effect on social participation and opportunity to control life circumstances. Psychologists have found the psychological effects produced by income disparities on groups and individuals in the population. These psychological theories hold that disparities in income and social standing create stresses that can eventually damage health [18,19]. Social scientists have found that individual- and group-level social relationships influence health either directly or through more proximal factors. When income differences are smaller, people are more trusting of one another and more likely to participate in communal activities, and this social cohesiveness is linked to lower overall mortality and better self-rated health [20,21]. Shi [22] found that there is relationship between stroke and income and that the impact of income inequality on stroke mortality was reduced in the presence of primary care.

Epidemiological studies have revealed that physical inactivity represents a high risk of premature development of chronic diseases such as cardiovascular diseases. Physical inactivity can increase the risk of high blood pressure, high blood cholesterol, diabetes, heart disease and stroke. Studies have found that greening of urban areas could make a contribution to increase physical activity [23]. International studies have documented positive health effects of green areas on human health (e.g., [15,24-28]. Using satellite data, Kimes [29] found that urban areas with the highest asthma hospitalization rates have the lowest vegetation cover. No studies have been done to investigate the association between stroke and greenness. Even though some studies have shown an association between green space and health, there is most likely also an association between local or accessible green space and socioeconomic status, e.g., income, race, and education, which may act as confounders. Another possible confounder is the function of green space as an air purifier. Trees, forest and grassland not only create feelings of relaxation and well-being and areas for recreation and physical activity, which promotes good health, but also can improve air quality by removing pollutants and particulate from the air.

**Figure 1:**
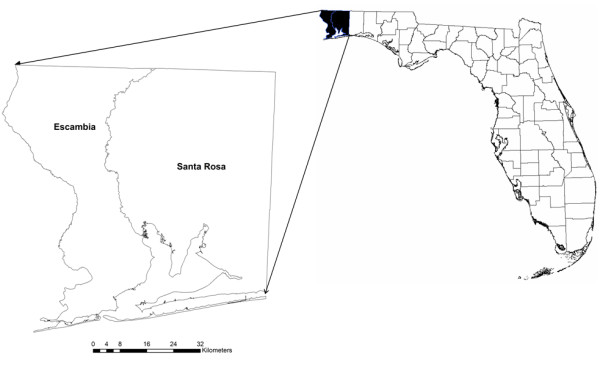
Study area.

Our study area includes Escambia and Santa Rosa counties in northwest Florida (Figure 1). According to the Florida CHARTS (Community Health Resources Tool Set) [30], in 2003, stroke age-adjusted death rate (per 100,000) is 53.5 for the US, 42.4 for Florida, 74.1 for Escambia County, and 50.0 for Santa Rosa County. Escambia County has much higher stroke death rate than the state and the country. The objective of this study was to examine if there are associations between stroke and air pollution, income and greenness in northwest Florida. Our study uses an ecological geographical approach. We collected stroke data at the census tract level. We derived raster surfaces of densities of both point and mobile air polluters. We calculated greenness using remotely sensed imagery. Dasymetric mapping technique was employed to calculate average values of air pollution and greenness variables for each census tract. We last adopted a Bayesian hierarchical model with a spatially structured random effect and a random effect with an exchangeable non-spatial prior distribution to link stroke date with air pollution, income and greenness.

## Methods

### Stroke mortality rate standardization

Stroke death count data at the census tract level was obtained from Florida CHARTS website. The data was compiled from Florida Vital Statistics in a 5-year (1998–2002) aggregate. To adjust for age effect, expected number of stroke deaths for each census tract was calculated using indirect standardization [31]. The calculated death count is the number of cases that would be expected in the study population if people in the study population contracted the disease at the same rate as people in the standard population. This study used the US South population as standard population. This census region includes three census divisions (South Atlantic, East South Central, and West South Central) with 16 states and the District of Columbia. The age-specific stroke morality rates from the standard population for 1999–2004 were obtained from the National Vital Statistics System. Standardized mortality rates (SMRs) were calculated by dividing the observed count by the expected value. The observed and expected death count data are inputs to the Bayesian hierarchical statistical model.

### Dasymetric mapping

Dasymetric mapping, introduced as a cartographic technique [32], utilizes ancillary information to internally redistribute variables within the limits of their tabulation zone so as to create subzones of relative homogeneity and thereby ensure that mapped discontinuities better reflect the true underlying geography. By facilitating the spatial refinement of aggregated data, dasymetric maps can better show, in the case of population statistics, the places where people actually reside. A dasymetric map is a variation of choropleth map. Choropleth mapping visually displays data by assigning the value of the variable of interest to the corresponding spatial unit and using different shades or textures for each value or classified values creating a thematic map. However, the phenomenon to be mapped is often not dispersed evenly within the spatial units, and choropleth mapping masks the underlying nuances of the distribution. Artificial boundaries, along with variations in the size of the unit of analysis, can, for instance, distort the true distribution of a phenomenon [33]. Dasymatric mapping provides a methodology for refining the distribution of a phenomenon within a spatial unit. Compared to choropleth maps, dasymetric maps are generally considered to provide more detailed and accurate description of the spatial pattern. In terms of its actual implementation, dasymetric mapping lacks a standardized methodology and variations are possible in terms of the choice of ancillary data used or the degree of internal differentiation attempted. Most applications of dasymetric mapping use a binary method (e.g. [34-36]). The binary dasymetric method consists of internally mapping each census tabulation zone into subareas identified as either occupied or empty, then allocating the population count to only the occupied portion.

**Figure 2:**
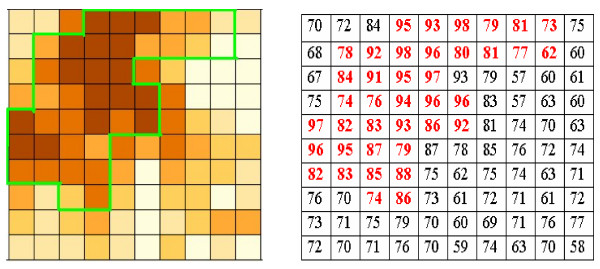
**Choropleth mapping vs. dasymetric mapping for environment exposure calculation**. Left: Map of an environment exposure (area within the green boundary represents human activity area and the whole square grid represents a census unit). Right: Cell values of the environment exposure (red represents human activity area). The mean exposure value is 87 for red cells, and 77 for the whole census unit.

Despite the many dasymetric mapping practices in demographic mapping for spatial representation of population density, population data areal interpolation, and crime analyses (e.g., [33-38]), none have been done in mapping environmental exposure variables. Human activities that expose people to air pollution are usually confined to developed area and road network. In this study, derivation of environment exposure variables from point and mobile source air polluters relies on calculation of average cell values of the variables within census enumerations districts on which stroke data are based. The conversion of the spatial support from point or line to area aggregation is necessary for linking disease data with environment exposure data. While in the city of Pensacola almost the whole census tract areas are occupied by people, most of the area in the northern rural region is sparsely inhabited. Average cell values of environment exposure variables based on choropleth mapping would be different from those based on dasymetric mapping. Figure 2 illustrates how average environment exposure values based on the two mapping methods are different. The left map in Figure 2 shows the spatial pattern of an environment exposure. Suppose that the whole square grid represents a census tract and the area within the green boundary is human activity area. The matrix of cell values is shown on the right with the red colour representing human activity area. The average exposure value based on the whole grid (choropleth mapping) is 77 while the average value calculated using human occupied cells (dasymetric mapping) is 87. For statistical modelling of disease and environment, it is reasonable to use the environment exposure value based on dasymetric mapping since people are exposed mostly to the environment where they live, work and travel.

For dasymetric mapping, a cloud-free Landsat 7 Enhanced Thematic Mapper Plus (ETM+) imagery acquired on November 22, 2000 was obtained from the Earth Resources Observation System Data Center. The image was classified using a decision tree algorithm. Developed areas were extracted to form a binary image with one to represent developed areas and zero for other areas. This binary image was further combined with the USGS 1998 road network map, and a 500 m buffer was built around the developed area and the roads. Selection of the buffer distance has considered normal human walking journey length away from where they live and work. This process results in a binary raster map showing human activity area and inhabited area. The map was further overlaid with the census tract map using GIS intersecting operation to extract human activity area within each tract for calculation of average exposure value for each tract.

### Air pollution data

To examine the relationship between stroke and air pollution, ideal air pollution data would be the individual exposure data. In an ecological study based on aggregate disease data, it is not practical to obtain individual exposure data. We might use monitored air quality data to represent ambient concentrations. The US EPA has set National Ambient Air Quality Standard (NAAQS) for six principal pollutants, which are called "criteria" pollutants: carbon monoxide (CO), lead (Pb), nitrogen dioxide (*NO*_2_), ozone (*O*_3_), particulate matter (PM), and sulfur dioxide (*SO*_2_). The concentrations of these criteria pollutants can be measured in the ambient air and we might use sample data from monitoring stations and GIS spatial interpolation techniques to derive a continuous concentration surface. However, in our study area, there are only two air monitoring sites located in the City of Pensacola, Escambia County. The sampling sites are not necessarily located in the areas with sources of high pollution. It did not seem prudent to use the monitored data to derive air quality raster surfaces for dasymetric mapping. Therefore, the monitored air quality measurements were not used to calculate exposure to air pollution. Instead, the maps of recorded sources of air pollution (both point sources and mobile sources) were used to derive polluter density surfaces as surrogates for ambient air pollution concentration.

Existing studies investigating the effect of air pollution on stroke have examined different criteria air pollutants. These studies lack consistency as to the presence of effects or, where the effects have been observed, the type of pollutant most responsible. Although there have been hypotheses about the potential mechanisms, the roles that specific air pollutants play in stroke remain unclear. In view of this fact, and due to unavailability of ambient concentration data, in this study, air pollutants are treated as a whole and the general effects of air pollution, rather than the effects of specific pollutants, are examined. A similar approach has been used in a study of asthma and air pollution in the Bronx, New York City by Maantay [39] where air pollution refers to the substances that constitute the pollutant mixture from traffic and industry-related sources that has been associated with respiratory effects. As to the question of what is counted as a hazard, Maantay [40] found that many of the studies focus on only one set of hazard facilities. However, studying the impacts of only one set of facilities produces misleading and incomplete results. Analysis must be able to take into account cumulative impacts from multiple sources of pollution and synergistic impacts from combining pollutants.

**Figure 3:**
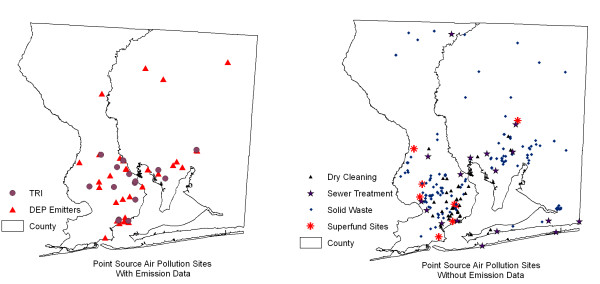
Maps of point source polluters.

**Figure 4:**
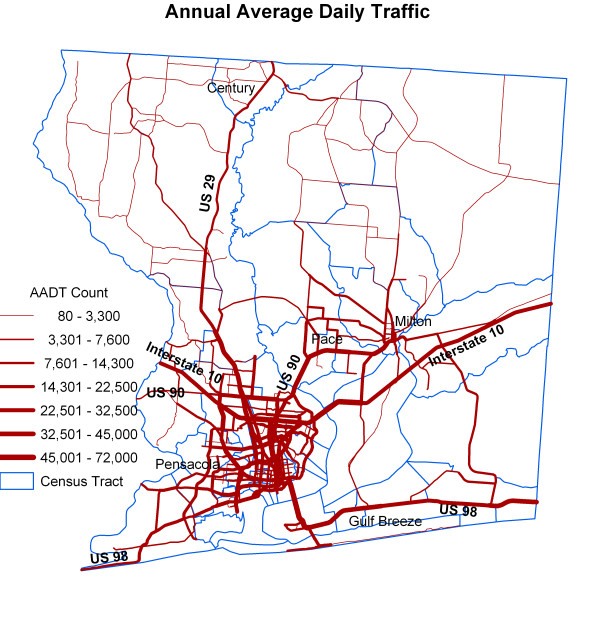
Annual average daily traffic count.

In our study, we considered air pollution from point locations where air emission data are derived from databases of US EPA (Toxic Release Inventory sites) and Florida Department of Environment Protection, and from other point source polluters where air emission data are not available which include sites of dry cleaning, sewer treatment, solid waste disposal, and Superfund sites (Figure 3). The date of the point source polluter distribution data coincides approximately with the time period of disease data. We also considered air pollution from vehicle traffic. For the mobile source pollution data, the annual average daily traffic (AADT) count data for road segments were obtained from Florida Department of Transportation (FDOT) (Figure 4). This dataset contains 1997 traffic data for the State of Florida from all portable and telemetered traffic meters. AADT is the total volume of traffic on a highway segment for one year divided by the total number of days in a year. Monitored roadways have at least one continuous counter in place for every functional class category, i.e., rural, principal arterial, etc., having at least 10 miles of road in one county. Major intersections and significant changes along the same roadway also are considered in the placement of counters. Where no counter is in place, information from the Road Characteristics Inventory [41] database is used to determine annual traffic volumes for this road segment (personal communication, Rick Reel, FDOT). In the study area, there are 539 road segments (Figure 4). The minimum AADT is 80, the maximum is 72,000, and the mean is 14,010. The minimum, maximum and average segment lengths are 88 m, 27,522 m, and 3,031 m respectively.

The statistical unit of disease data for this study is census tract. We might be able to design a surrogate variable to represent the exposure to total air pollution using either spatial coincidence methods or proximity to air polluters, as in many publications (see [40]). In a spatial coincidence method, we could count the number of point polluters within each census tract, and then calculate the number of polluters per unit area for each tract. However, close examination of the pollution source maps revealed that some polluters are located near census tract boundaries. Air pollution from these point sources has impact not only on people living in the tract containing the points, but also the neighbouring tract. A spatial coincidence method can not capture this effect. The resulting map will show a crisp boundary between tracts. In fact, the artificial administrative boundaries do not constitute a barrier to prevent air pollution from spreading from one tract to another. What we want is a continuous pollution surface. In other existing studies actual proximity to the air polluters is taken into account by either constructing buffer zones of the specified distances around the nearest polluter or just using the distance to the nearest point polluter. This method has a disadvantage that it considers the air pollution effect from only one point. In fact, the level of air pollution should be a composite effect of a group of polluters.

For our study, we used air polluter density surfaces to derive surrogate air pollution exposures. For calculation of density surfaces for point source polluters, we also included data from the counties neighbouring the study area to reduce the edge effect. The individual air polluter density surfaces were kept separate and were not combined into one pollution surface. The density surfaces were further used to extract aggregate zonal statistics (average density) based on dasymetric mapping. For the Florida DEP monitored polluters and the US EPA TRI sites with emission data, kernel density was calculated (kernel size = 6,000 m, the average distance between the points). The value of the total annual air emission item determines the number of times to count the point. Kernel density calculated the density of point features around each output raster cell (cell size = 30 m, the resolution of the satellite imagery). A smoothly curved surface was fitted over each point. The surface value is highest at the location of the point and diminishes with increasing distance from the point, reaching 0 at the search radius distance from the point. The volume under the surface equals the total air emission for the point. The density at each output raster cell was calculated by adding the values of all the kernel surfaces where they overlay the raster cell centre. The kernel function was based on the quadratic kernel function described by Silverman [42]. Maantay [40] found that many spatial analyses assume that one TRI facility, for instance, is equivalent to any other. But amounts of toxic emissions vary widely among facilities, and emission levels and toxicity are often not mapped or factored into the analysis. The advantages of the kernel density approach are that it has considered the amount of emissions and has factored in the effects from multiple point sources.

For point polluters (dry cleaning, sewer treatment, solid waste disposal, and Superfund sites) without emission data, simple point density surfaces were calculated. This procedure calculated the density of point features around each output raster cell. A circular neighbourhood was defined around each raster cell center, and the number of points that fall within the neighbourhood was totaled and divided by the area of the neighbourhood.

**Figure 5:**
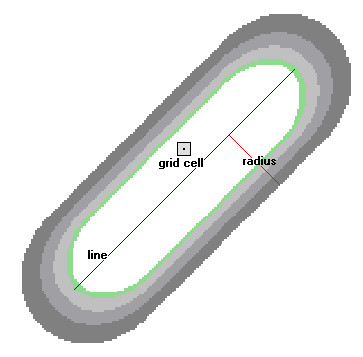
**How kernel density for line features works**. The illustration shows a line segment and the kernel surface fitted over it. The contribution of the line segment to density is equal to the value of the kernel surface at the raster cell center.

To derive an index representing traffic impact, a kernel density surface was calculated based on the traffic count data *AADT*. A smooth, curved surface was fitted over each road segment [43]. The surface value is greatest when it is on the line and diminishes as it moves away from the line, reaching zero at the search radius distance (2.5 km) from the line. The surface was defined so that the volume under the surface equals the product of line length and the AADT value. In Figure 5, a road segment is displayed with a kernel surface fitted over it. The contribution of this segment to density equals the value of the kernel surface at the raster cell center. The density at each output raster cell was calculated by adding the values of all the kernel surfaces where they overlay the raster cell center. The use of the kernel function for roads was also adapted from the quadratic kernel function as described in Silverman [42].

### Greenness

To investigate the impact of greenness on health indicators, Nielsen and Hansen [28] used data derived from a questionnaire which includes questions on the distance from the home to different types of green and recreational areas such as parks, forests, beaches, green sports facilities, green residential areas and the like, and the frequency of visits to these areas. Satellite remote sensing with its synoptic view of the earth's surface, regular repetitive coverage over a large area, and digital mode of data capture offers an effective means in measuring our natural environment. Our study derived a 'greenness' GIS layer to represent the amount of exposure to green space using the Landsat 7 ETM+ imagery and tasseled cap transformation [44]. It should be noted that the existence of green space is not necessarily equivalent to access to it (in terms of either physical or cultural accessibility). If green space can have a health benefit as a function of increased physical activity, not all green spaces are the same. An inaccessible forest near the highway would not necessarily offer the same opportunity for physical activity as a park or soccer field. However, in terms of the function of the green spaces as an air purifier and stress reducer, the issue of accessibility of different types of green spaces (e.g., public/closed parks, forests, etc.) might not be so worthy of consideration. Even though a green space is inaccessible and thus does not increase physical activity, it could improve air quality for the local neighborhood and provide aesthetic appearance and feelings of relaxation for people at a distance from the green scene.

### Bayesian hierarchical modelling of relationship between stroke and income and environment exposure

We used a Bayesian hierarchical model to explore the association between stroke mortality and point and mobile source air pollution as well as income and greenness. Bayesian hierarchical models have been widely used in the field of disease mapping [45-47]. Simulation-based algorithms for Bayesian inference allow us to fit very complicated hierarchical models, including those with spatially correlated random effects. In this geographical and ecological study, there could exist spatial autocorrelation within values of the stroke mortality and air pollution variables. We fitted the following model, allowing a convolution prior for the random effects:

*O*_*i *_~ *Poisson*(*μ*_*i*_)

log *μ*_*i *_= log *E*_*i *_+ *β*_0 _+ *β*_1_*INC*_*i*_/10000 + *β*_2_*AADT*_*i*_/10000 + *β*_3_*EPNT*_*i *_+ *β*_4_*PPNT*_*i *_+ *β*_5_*GREEN*/10 + *b*_*i *_+ *h*_*i*_

where *i *is the index for a census tract (*i *= 1,2, ..., 77), *O *is observed stroke death count, *E *is expected death count reflecting age-standardized values. We considered five census tract specific covariates: household income (*INC*) which uses the year 2000 census data, *AADT*, emission density of monitored point source polluters (*EPNT*), point density of point source polluters without emission data (*PPNT*), and greenness (*GREEN*). It should be noted that income and greenness might not be sufficiently independent. There is likely a strong relationship between greenness, or more accurately access to green or open spaces, and income. The possible relationship could cause the issue of collinearity in Bayesian modeling.

For model specification, we assumed an improper (flat) prior for the intercept parameter *β*_0 _and uniform prior distributions for the fixed-effect parameters (*β*). By fixed effects we mean they apply equally to all the census units. We included two sets of tract-specific random effects. The first set *b*_*i *_is spatially structured random effects assigned an *intrinsic Gaussian conditional auto-regression (CAR) prior *distribution [48]. The second set of random effects *h*_*i *_is assigned an exchangeable (non-spatial) *Normal *prior. The random effect for each census tract is thus the sum of a spatially structured component *b*_*i *_and an unstructured component *h*_*i*_. This is termed a convolution prior [48,49]. The model is more flexible than assuming only *CAR *random effects, since it allows the data to decide how much of the residual disease risk is due to spatially structured variation, and how much is unstructured over-dispersion. To complete the model specification, we assigned conjugate inverse-gamma prior distributions to the variance parameters associated with the exchangeable and/or *CAR *priors. More specifically, we defined the hyperpriors

$$\frac{1}{\upsilon_{\psi }}\sim \gamma (0.5,0.0005)$$

and

$$\frac{1}{\upsilon_{CAR}}\sim \gamma (0.5,0.0005).$$

We used the Markov chain Monte Carlo (MCMC) simulation computation technique and Gibbs sampling algorithm to fit the Bayesian model. Having specified the model as a full joint distribution on all quantities, whether parameters or observables, we wish to sample values of the unknown parameters from their conditional (posterior) distribution given those stochastic nodes that have been observed. MCMC methods perform Monte Carlo simulations generating parameter values from Markov chains having stationary distributions identical to the joint posterior distribution of interest. After these Markov chains converge to a stationary distribution, the simulated parameter values represent a correlated sample of observations from the posterior distribution. The basic idea behind the Gibbs sampling algorithm is to successively sample from the conditional distribution of each node given all the others. Under broad conditions this process eventually provides samples from the joint posterior distribution of the unknown quantities. Summaries of the post-convergence MCMC samples provide posterior inference for model parameters. The MCMC computation techniques and Bayesian analysis allow us to use more elaborate models without conventional model restrictions. The model also allows us to treat the stroke death counts as outcomes without the awkward transformations of the outcome and covariates of interest required to meet standard assumptions for linear regression models. The result of such analysis is the posterior distribution of an intensity function with covariate effects.

The model was fitted using the *WinBUGS *software – an interactive Windows version of the *BUGS *(**B**ayesian inference **U**sing **G**ibbs **S**ampling) program for Bayesian analysis of complex statistical models using MCMC techniques [50]. A spatial adjacency matrix (*w*_*ij *_= 1 when census tract *i *and *j *share a boundary, *w*_*ij *_= 0 otherwise) that is required as input for the conditional autoregressive distribution was created from the census tract map using GeoBUGS, an add-on module to WinBUGS. Typical disease mapping applications consider adjacency-based weights. Other weighting options (e.g., distance-decay weights) also appear in the literature (e.g., [51]) but are much less widely applied. Two Markov chains were simulated in the present study. The MCMC samplers were given initial values for each stochastic node. A total of 10,000 iterations with 5,000 burn-in was run. "Burn-in" denotes iterations that were discarded due to non-convergence of the model at the early stages of the algorithm. A visual inspection of the Markov chain trace plots of the sample values versus iteration has indicated that convergence has been reached after 5,000 iterations. We based our inference on iterations 5,001 to 10,000.

In addition to inference for the fixed effects, we also used the MCMC samples to explore patterns in the local standardized mortality rates (SMRs). The modelled local SMR is relative risk (RR) which incorporates fixed and random effects. The RR map provides insight into differences between the modelled expected stroke death count values and the *E*_*i *_values that do not adjust for covariate effects or regional differences. More specifically, we considered inference for *RR*_*i *_= 100 * exp(*β*_0 _+ *β*_1_*INC*_*i*_/10000 + *β*_2_*AADT*_*i*_/10000 + *β*_3_*EPNT*_*i *_+ *β*_4_*PPNT*_*i *_+ *β*_5_*GREEN*/10 + *b*_*i *_+ *h*_*i*_), where we multiplied by 100 to convert the local SMRs to their traditional scale. In our MCMC-based Bayesian analysis, calculating

$$RR_{i}^{n}=100*\exp(\beta_{0}^{n}+\beta_{1}^{n}INC_{i}/10000+\beta_{2}^{n}AADT_{i}+\beta_{3}^{n}EPNT_{i}+\beta_{4}^{n}PPNT_{i}+\beta_{5}^{n}GREEN/10+b_{i}^{n}+h_{i}^{n})$$

for each iteration *n *of the postconvergence MCMC samples (where *n *denotes the *n*^*th *^simulated value for each parameter), we obtained a sample from the posterior distribution of each of the 77 local SMRs.

## Results

**Figure 6:**
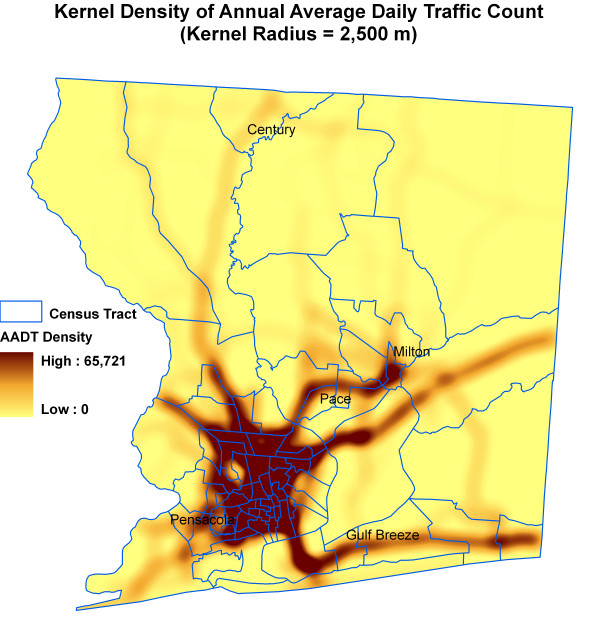
Raster surface of kernel density of annual average daily traffic count.

**Figure 7:**
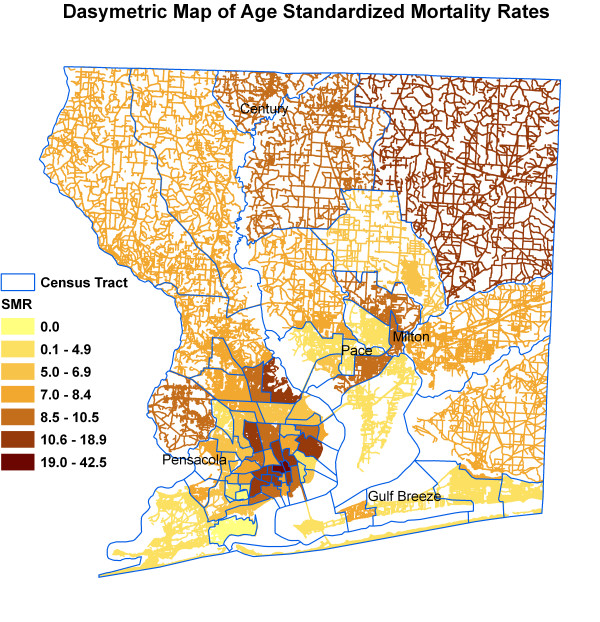
Dasymetric map of SMRs.

**Figure 8:**
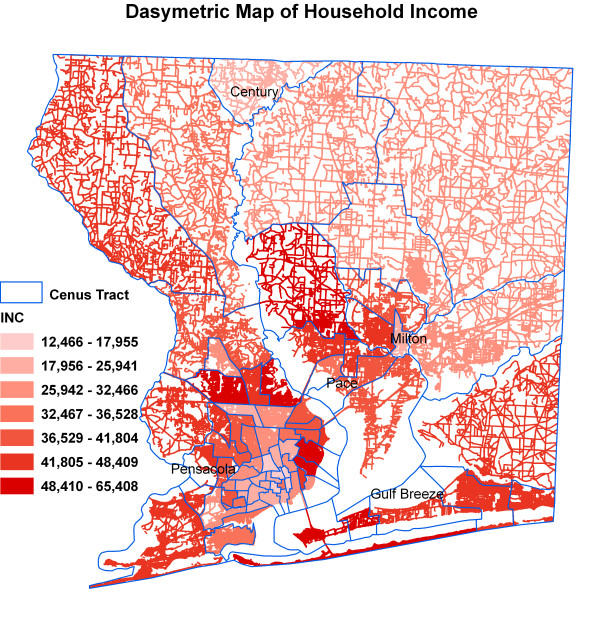
Dasymetric map of household income.

**Figure 9:**
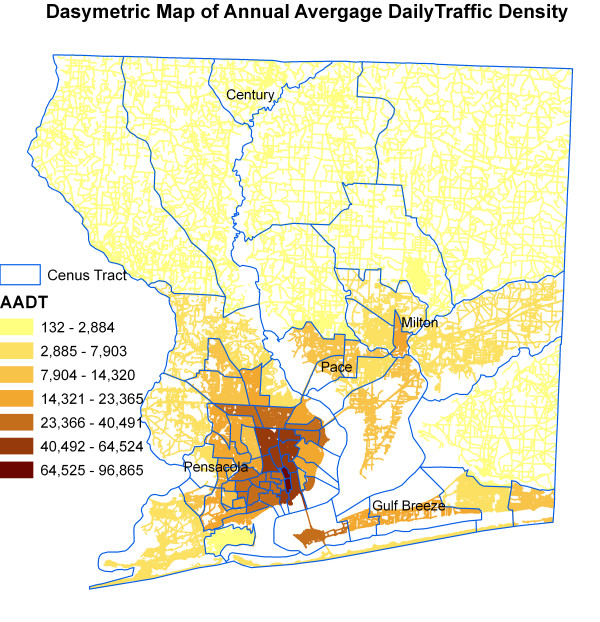
Dasymetric map of annual average daily traffic density.

**Figure 10:**
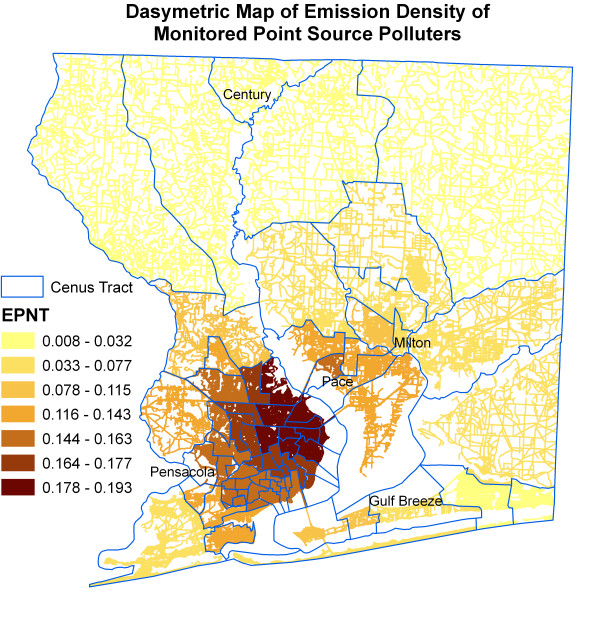
Dasymetric map of emission density of monitored point source polluters.

**Figure 11:**
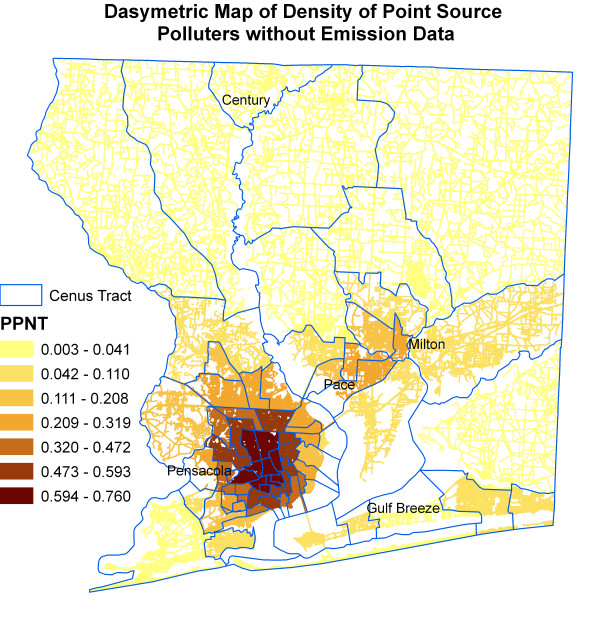
Dasymetric map of density of point source polluters without emission data.

**Figure 12:**
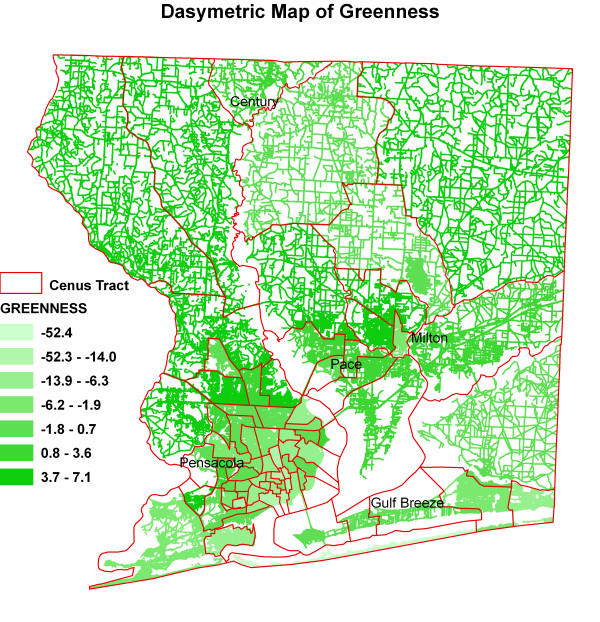
Dasymetric map of greenness.

Figure 6 shows kernel density surface of annual average daily traffic count. Figures 7, 8, 9, 10, 11, and 12 show dasymetric maps of the age-standardized stroke mortality rates (SMRs), income, and derived raster density surfaces for air pollution and greenness. The minimum, mean, and maximum SMRs are 0, 8.39, and 42.4 respectively. The mean age-adjusted stroke death rates were 8.39 times the average age-adjusted stroke rate in the US South. A quick visual inspection of the map patterns reveals that high stroke mortality rates concentrate in areas with low income, low greenness, and high air pollution.

**Figure 13:**
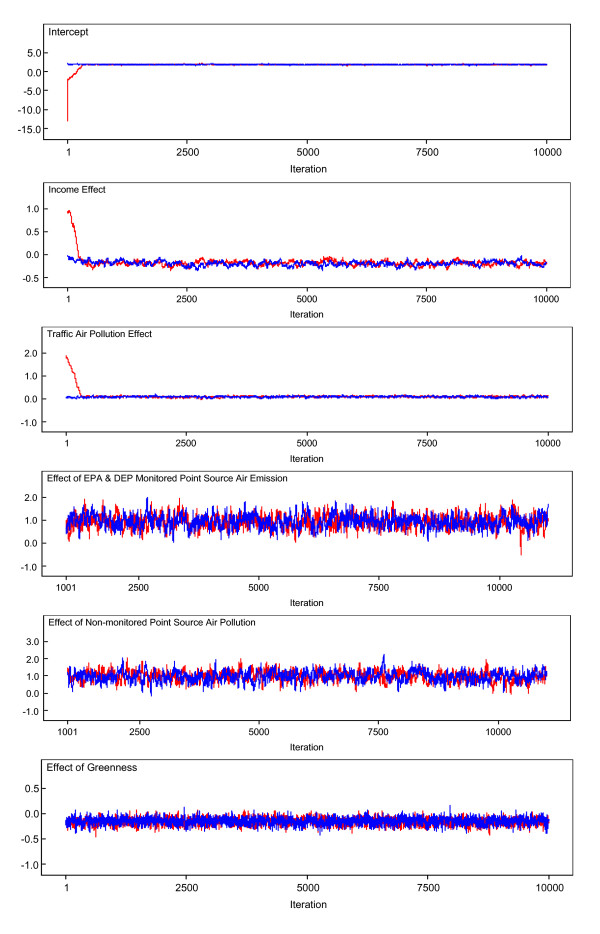
**Trace plots of the 10,000 Markov Chain Monte Carlo (MCMC) updates**. Simulation trace plots for the intercept, income effect, traffic air pollution effect, effect of EPA and Florida DEP monitored point source air emission, effect of non-monitored point source air pollution, and greenness effect for the Bayesian hierarchical model with a convolution prior. Horizontal axis represents iteration number and vertical axis represents simulated parameter value. The red trace is for one Markov chain, and the blue for the other.

Figure 13 illustrates a trace of the 10,000 updates of the fixed effects in each of model parameters in *β *= (*β*_0_, *β*_1_, *β*_2_, *β*_3_, *β*_4_, *β*_5_)'. The horizontal axis represents the simulation iteration number. The vertical axis represents simulated parameter values. Positive parameter values indicate positive relationship of the variable with stroke mortality. Negative values indicate a negative effect. The red trace is for one Markov chain, and the blue for the other. For each parameter, we observed the simulation move away from the initial seed values and then generate values fluctuating around within a consistent range of values representing the posterior distribution of each model parameter, indicating that convergence has been reached.

Inferences were made about the parameters of the model using the simulated values on iterations 5,001 to 10,000. Table 1 provides the estimated posterior mean, median, and associated 95% credible set for each of the fixed effects. A 95% credible set defines an interval having a 0.95 posterior probability of containing the parameter of interest which is assumed to be a random variable in Bayesian statistics. The 2.5%, 50% (median) and 97.5% quantiles and posterior mean were calculated via an approximate algorithm [52]. Summaries of the postconvergence MCMC samples provide posterior inference for model parameters. For example, the sample mean of the postconvergence sampled values for a particular model parameter provides an estimate of the marginal posterior mean and a point estimate of the parameter itself. The interval defined by the 2.5^th ^and 97.5^th ^quantiles of the postconvergence sampled values for a model parameter provides a 95% interval estimate of the parameter. In Bayesian inference, such an interval is termed a *credible set *to distinguish it from the *confidence interval *of classical statistics. Although similar in spirit, the interpretation of the two intervals is different [53]. A 95% credible set defines an interval having a 0.95 posterior probability of containing the parameter of interest (which is assumed to be a random variable in Bayesian statistics). In contrast, a 95% confidence interval represents an interval such that 95% of intervals constructed similarly from identically distributed and independent data sets would contain the true parameter value (which is assumed to be a fixed but unknown quantity in classical statistics).

**Table 1:** Markov chain Monte Carlo results for Bayesian hierarchical modelling of stroke mortality vs. income, air pollution, and greenness*

Fixed Effects	Posterior Mean	Posterior Median	Standard Deviation	MC Error	95% Credible Set
*β*_0_	1.829	1.832	0.083	0.004	(1.661, 1.986)
*β*_1_	-0.193	-0.193	0.047	0.003	(-0.286, -0.097)
*β*_2_	0.089	0.089	0.028	0.001	(0.034, 0.144)
*β*_3_	0.937	0.932	0.276	0.010	(0.419, 1.495)
*β*_4_	0.974	0.980	0.290	0.012	(0.413, 1.522)
*β*_5_	-0.161	-0.161	0.067	0.002	(-0.289,-0.031)

* Posterior means, medians, and 95% credible sets are based on 5,000 postconvergence iterations (from 5,001 to 10,000). Fixed effects are: *β*_0 _- intercept, *β*_1 _- income effect, *β*_2 _- traffic air pollution effect, *β*_3 _- effect of EPA and Florida DEP monitored point source air emission, *β*_4 _- effect of non-monitored point source air pollution, and *β*_5 _- greenness.

**Figure 14:**
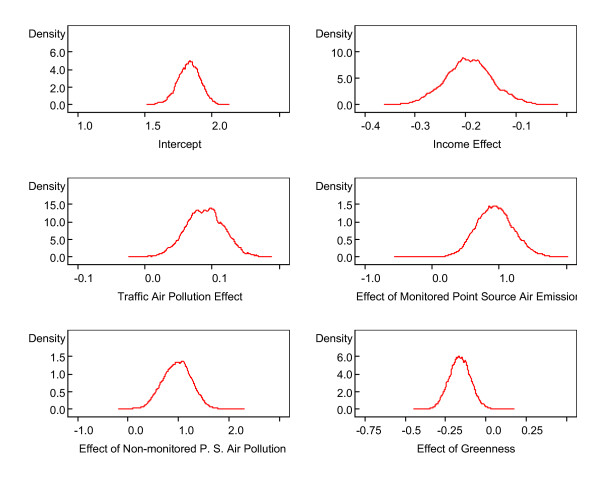
**Kernel estimates of the posterior densities of the fixed effects in the Bayesian hierarchical model**. Horizontal axis represents simulated parameter values and vertical axis represents the density of each value.

Standard deviations and Monte Carlo (MC) errors were calculated. One way to assess the accuracy of the simulation is by calculating the MC error for each parameter. This is an estimate of the difference between the mean of the sampled values (which we were using as our estimate of the posterior mean for each parameter) and the true posterior mean. As a rule of thumb, the simulation should be run until the Monte Carlo error for each parameter of interest is less than about 5% of the sample standard deviation. The MC errors calculated from the iterations 5,000 to 10,000 for all the parameters are less than 5% of the corresponding standard deviations, suggesting an accurate posterior estimate for each parameter. In addition, Figure 14 provides kernel estimates of the corresponding posterior densities. The horizontal axis represents simulated parameter value. The vertical axis represents the density of each parameter value. From the results in Table 1 and Figure 14, we note strong negative effects of income and greenness (the posterior densities of *β*_1 _and *β*_5 _primarily cover negative values) and positive effects of both mobile and point source air pollution (the 95% credible sets cover positive values).

**Figure 15:**
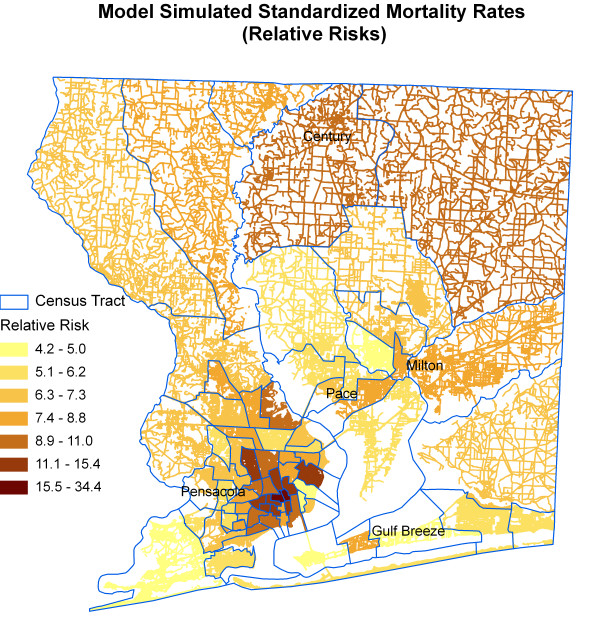
Model simulated standardized mortality rates (relative risks).

Figure 15 shows a map of the posterior median value of *SMR*_*i *_for each census tract. The minimum, mean, and maximum median values of *SMR*_*i *_are 4.22, 8.06, and 34.42 respectively. We note concentrations of high SMRs in and around Pensacola city and north of Pensacola. Compare this map with the age standardized mortality map in Figure 7. The relative risk map incorporating covariates does not show an obvious concentration of high SMR in Pace and Milton area as in the SMR map adjusting for age only. Census tracts in the north and northeastern parts of Santa Rosa County also exhibit large local SMRs. In fact, these areas have low exposure to air pollution. This odd behavior is due primarily to low local population sizes.

## Discussion

We found an excess risk of stroke mortality in areas with high air pollution levels. Several potential mechanisms have been proposed to explain the association between air pollution and stroke. It has been hypothesized that fine particulate air pollution provokes alveolar inflammation, causing the release of potentially harmful cytokines, which results in increased coagulability [54]. Experimental evidence suggests that ultrafine particles are able to penetrate the human lung and enter the blood circulation [55]. Particulate air pollution has been shown to induce progression of atherosclerosis in an animal model [56]. We also found a negative effect of income level, i.e., higher risk of stroke mortality occurs in areas with lower income. It is interesting to find that exposure to more green space could reduce the risk of stroke mortality. The empirical result in this study supports the "salutary" effects of the exposure to natural environments on human health [57] as well as the significance of residential environments to counteract "sedentary" lifestyles [58] and that greening of urban areas could make a contribution to increase physical activity [23].

There are several strengths in our study. The first is the use of ecological approach and the Bayesian hierarchical model. Ecological studies are particularly useful when the individual level of exposure is either difficult or impossible to obtain, or can be only measured imprecisely [51]. An advantage to performing an ecological analysis is that we were able to control for unmeasured ecological-level confounders. In our study, we were able to include income and greenness as covariates in addition to air pollution. Ecological studies are more useful for generating and testing hypothesis (i.e. qualitative identification of an association) rather than quantitative estimation of the strength of an exposure-response relationship [59]. The Bayesian model has advantages over conventional statistical models in that it has the ability to deal with the issue of spatial autocorrelation by incorporating a spatially structured random effect. It allows us to deal with extra-Poisson variation exacerbated by the small scale as well as to take into account various area-specific covariates. Secondly, the use of kernel density approach was able to consider amount of emissions and cumulative impacts from multiple sources of air pollution. Thirdly, we adopted dasymetric mapping method to calculate aggregate environment exposures whose values are based on human activity areas only. This has improved data accuracy and reliability of the model results. Lastly, to our knowledge, the present study is the first to investigate the association of stroke with greenness. We took advantage of satellite remote sensing for our environment health study.

In interpreting the results of this study, several limitations require consideration. We did not examine ischemic and hemorrhagic stroke separately due to unavailability of the data at the census tract level. Some studies have shown that the effects of air pollutants on ischemic stroke mortality were statistically significant, whereas this was not the case for hemorrhagic stroke mortality [7,60]. The use of polluter density data does not necessarily represent individual exposure. The use of aggregated data and therefore inferences based on the analysis cannot be directly transferred to the individual level. We did not consider ethnicity effect. Although it is important to construct a model of health determinants that include relevant risk factors, many of these factors are collinear and therefore cannot be included in the same model. Race and income are inextricably linked and an effort to isolate class and race makes little sense [61]. In our study using an ordinary least regression model and the US Census 2000 data at the tract level, we found that there is a significant negative relationship between income and race (*p *< 0.001). Census tracts with a higher proportion of black residents (including Hispanic blacks) have a lower income level. An inherent limitation of an ecological geographic study is that it uses aggregate data and does not have the ability to incorporate individual information, e.g., individual migration, time length of residence, and the separation of places of work, recreation and living. We selected a time frame for data based on what was available. Our analysis did not consider the lag-time between a potential exposure and the occurrence of the disease symptoms. People may have been exposed much earlier and might have lived at a different place than where the first signs of the disease occur and where they die. For dasymetric mapping, it is the residents of the census tract being disaggregated that are redistributed to the developed areas. The assumption behind this is that these areas are equivalent to spaces of human activity used by those that reside within that specific census tract. Since the developed areas also include road networks and parking lots, this will not always be the case (e.g. the roads and parking will likely be used by those from other geographies). Although the use of polluter density surfaces has advantages over spatial coincidence and spatial proximity indices in that it takes into account the amount of emission and accumulative effects from multiple pollution sources, it shares the same drawback of inability to consider spatial heterogeneity of the spread of air pollution due to variance in location relative to polluters, wind direction, terrain, and many other factors. Also, the TRI emission quantities are estimated and not measured amounts and we were not able to consider different types of pollutants. Our future research will have to use air dispersion modelling to derive concentrations of criteria air pollutants. We are also interested in merging aerosol optical depth satellite data with ground monitor data to derive concentration surfaces of particulate matter.

## Conclusion

Our ecological study using dasymetric mapping and Bayesian hierarchical modelling for Escamiba and Santa Rosa Counties in northwest Florida has found association between stroke mortality risk and household income, air pollution from both traffic and point source polluters, and greenness. High risk of stroke mortality was found in areas with low income level, high air pollution level, and low level of exposure to green space. The findings of the study point to the issues of environmental injustice, socioeconomic injustice and health inequality. A green health perspective in urban planning could have an important role in future disease prevention and health promotion activities. The linkage between stroke and air pollution suggests to policy-makers that targeting policy interventions at high pollution areas may be a feasible option for stroke prevention.

## Competing interests

The authors declare that they have no competing interests.

## Authors' contributions

ZH conceived of the study, conducted the research and wrote the manuscript. JL and KRR revised the manuscript. All authors read and approved the final manuscript.
